# DTMS Combined with a Pain-directed Psychotherapeutic Intervention in Fibromyalgia - A Randomized Double-blind Sham-controlled Study

**DOI:** 10.1192/j.eurpsy.2023.571

**Published:** 2023-07-19

**Authors:** E. Tilbor, A. Hadar, V. Portnoy, O. Ganor, Y. Braw, H. Amital, J. Ablin, C. Dror, Y. Bloch, U. Nitzan

**Affiliations:** ^1^Shalvata Mental Health Center, Hod Hasharon; ^2^Neuropsychology Laboratory, Ariel University, Ariel; ^3^Sheba Medical Center, Ramat Gan; ^4^Sourasky Medical Center, Tel Aviv, Israel

## Abstract

**Introduction:**

Fibromyalgia Syndrome (FMS) is a highly prevalent condition, causing chronic pain and severe reduction in quality of life and productivity, as well as social isolation (Birtane *et al.* Clinical Rheumatology 2007; 26(5), pp. 679–684; Arnold *et al.* Psychosomatics. England 2010; 51(6), pp. 489–497; Lacasse, Bourgault and Choinière. BMC Musculoskeletal Disorders 2016; 17(1), pp. 1–9). Despite significant morbidity and economic burden caused by FMS, current treatments are scarce (Busch *et al.* The Journal of rheumatology. Canada 2008; 35(6), pp. 1130–1144; Bernardy *et al.* Journal of Rheumatology 2010; 37(10), pp. 1991–2005; Jackson *et al.* American journal of hematology 2016; 91(5), pp. 476–80).

**Objectives:**

To examine whether stimulation of the dorsal Anterior-Cingulate-Cortex and the medial Prefrontal-Cortex (ACC-mPFC) activity by deep Transcranial Magnetic Stimulation (dTMS) enhances a pain-directed psychotherapeutic intervention.

**Methods:**

Nineteen FMS patients were randomized to either 20 sessions of dTMS or sham stimulation, each followed by a pain-directed psychotherapeutic intervention. Using H7 HAC-coil or sham stimulation, we targeted the ACC-mPFC; specific brain areas that have a central role in pain processing (Fomberstein, Qadri and Ramani. Current Opinion in Anaesthesiology 2013; 26(5), pp. 588–593; Tendler, A. *et al.* Expert Review of Medical Devices 2016; 13(10), pp. 987–1000). Clinical response to treatment was evaluated using the McGill Pain Questionnaire (MPQ), Visual Analogue Fibromyalgia Impact Questionnaire (VAS-FIQ), Brief Pain Inventory questionnaire (BPI), and the Hamilton Depression Rating Scale (HDRS).

**Results:**

DTMS treatment was safe and well tolerated by FMS patients. A significant decrease in the sensory and affective pain dimensions was demonstrated specifically in the dTMS cohort, as measured by the MPQ using paired-sample t-tests with Bonferroni correction for multiple comparisons on three-time points (Significant group x time interaction [*F(2, 34) = 3.79, p < .05*, η^2 =^ 0.183]. No significant changes were found in the cognitive functions, psychophysical measurements of pain, or depressive symptoms in both dTMS and sham groups and between groups.

**Conclusions:**

Our findings suggest that a course of dTMS combined with a pain-directed psychotherapeutic intervention can alleviate pain symptoms in FMS patients. Beyond the clinical possibilities, future studies are needed to substantiate the innovative hypothesis that it is not the dTMS alone, but rather dTMS driven plasticity of pain-related networks, that enables the efficacy of pain-directed psychotherapeutic interventions.

**Disclosure of Interest:**

None Declared

